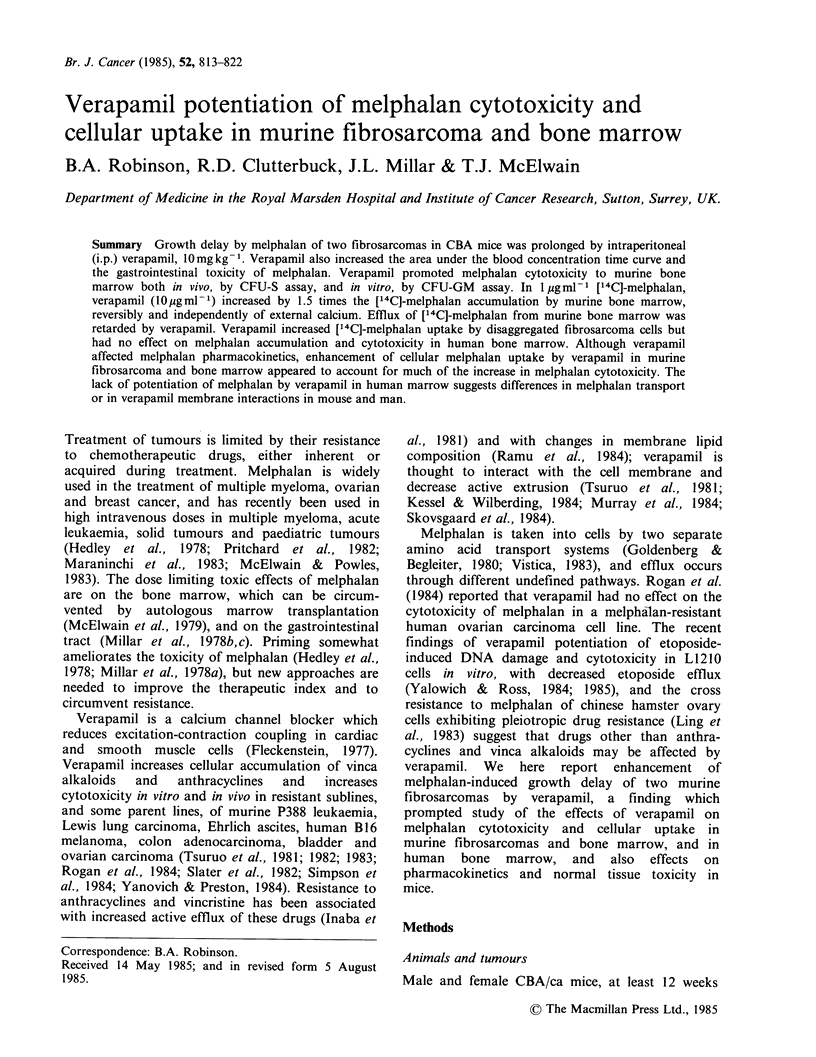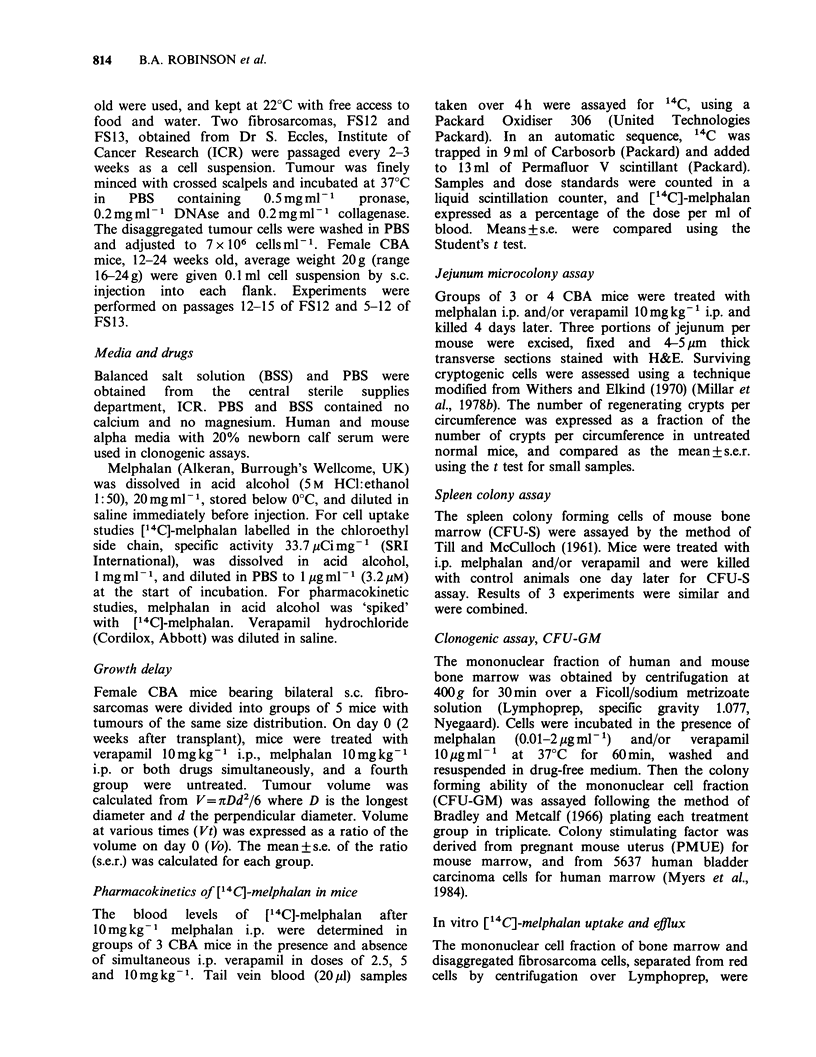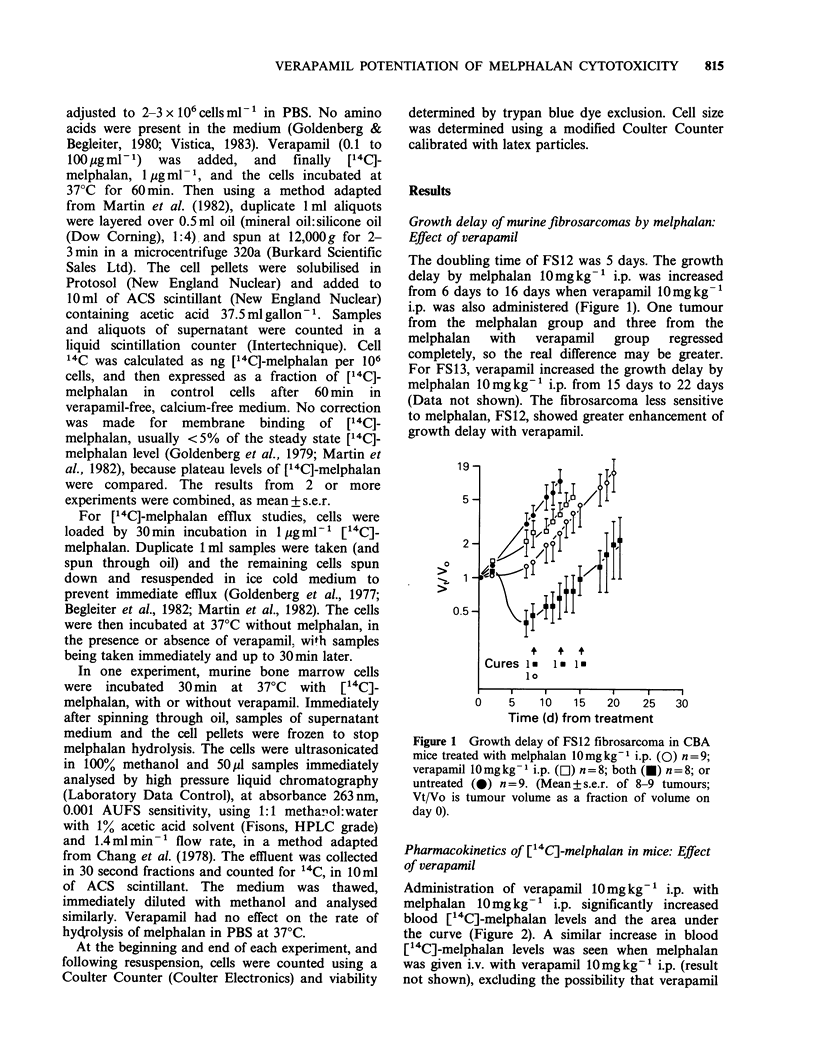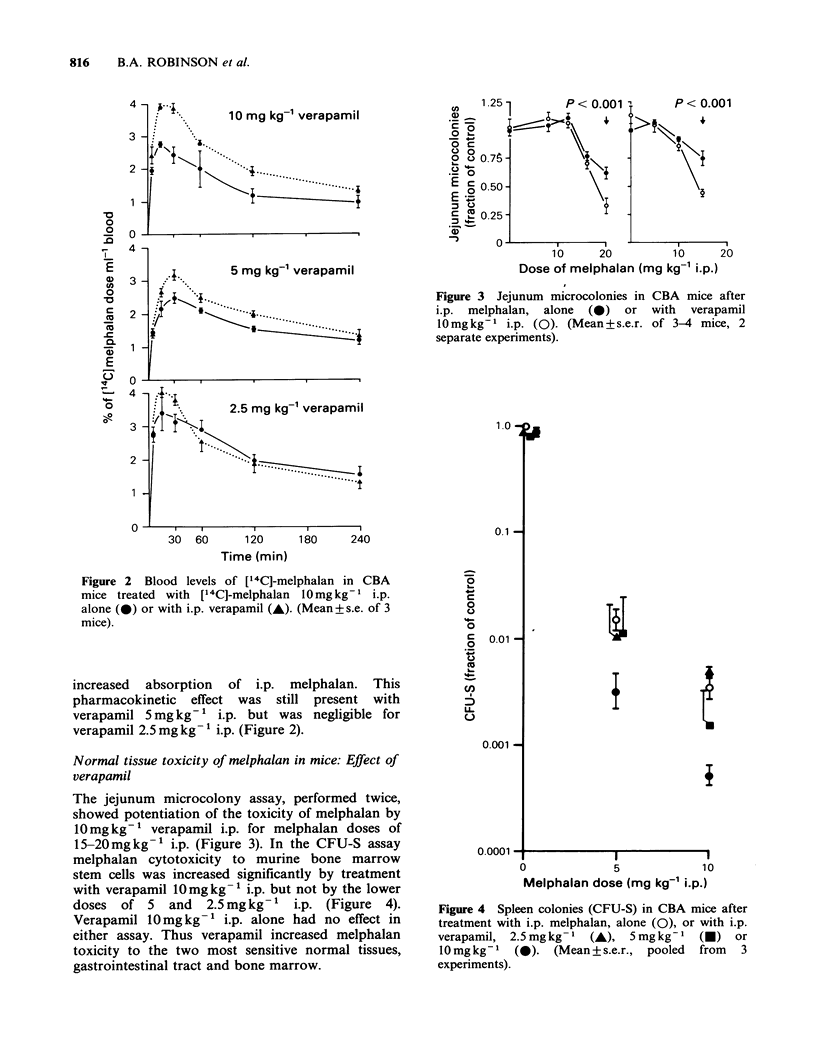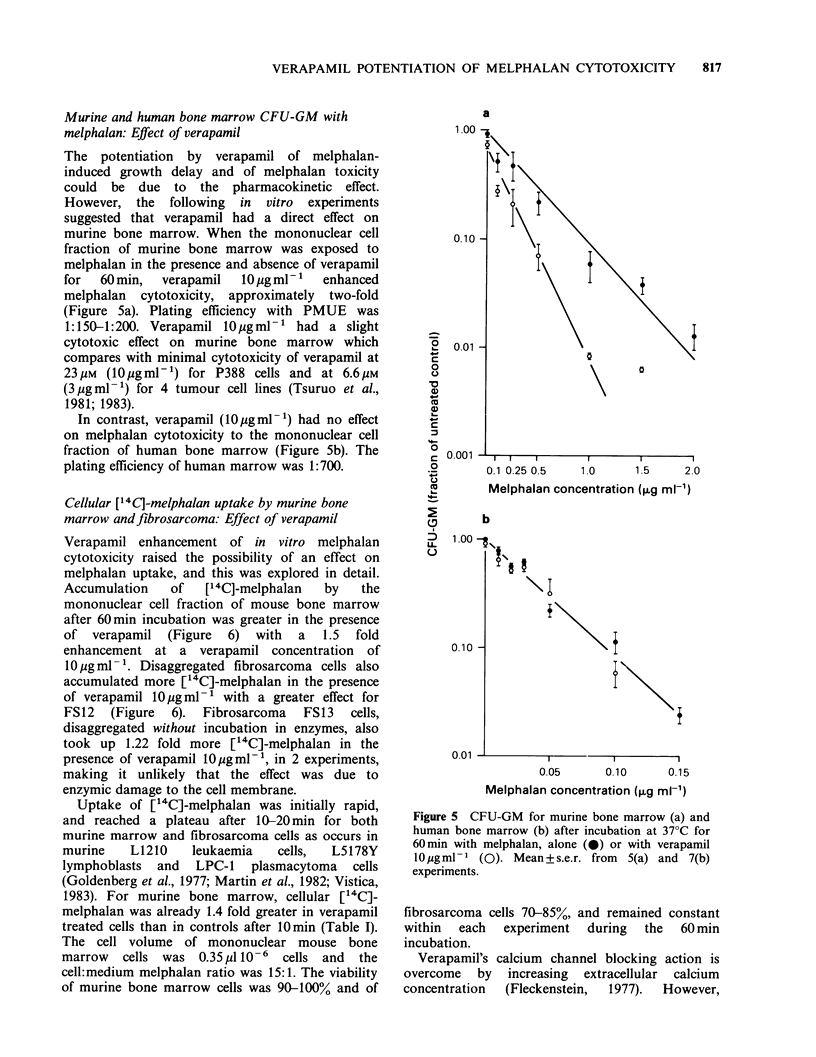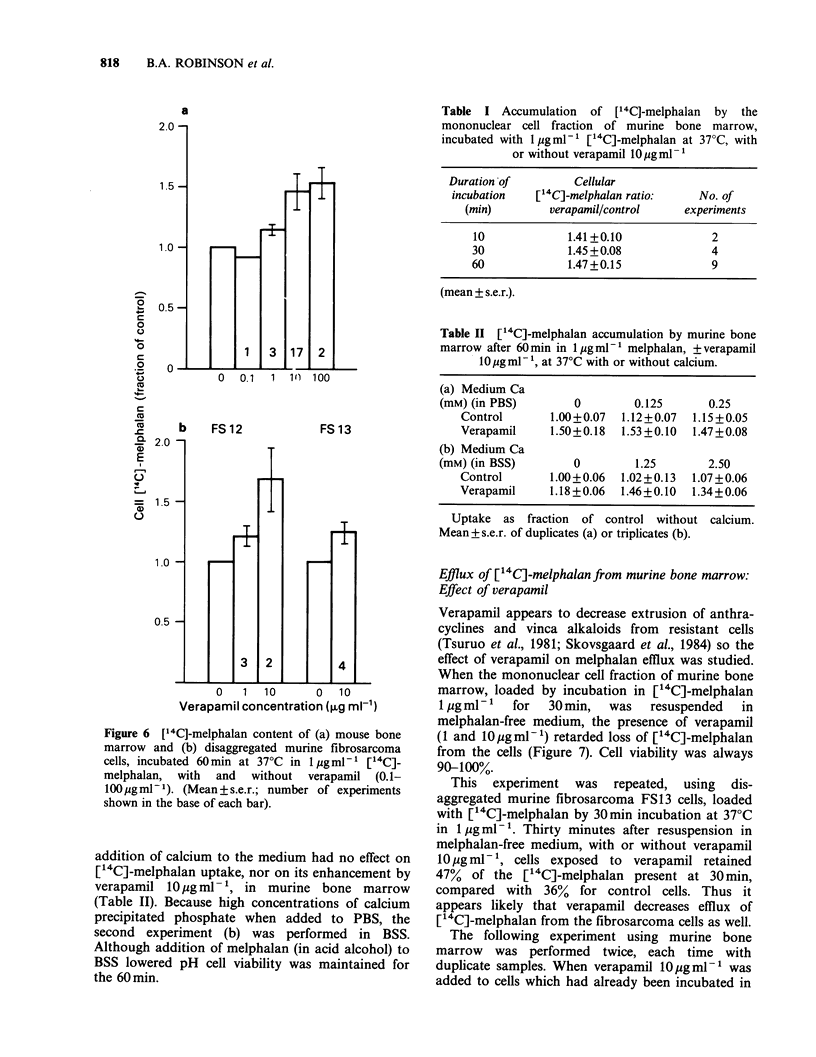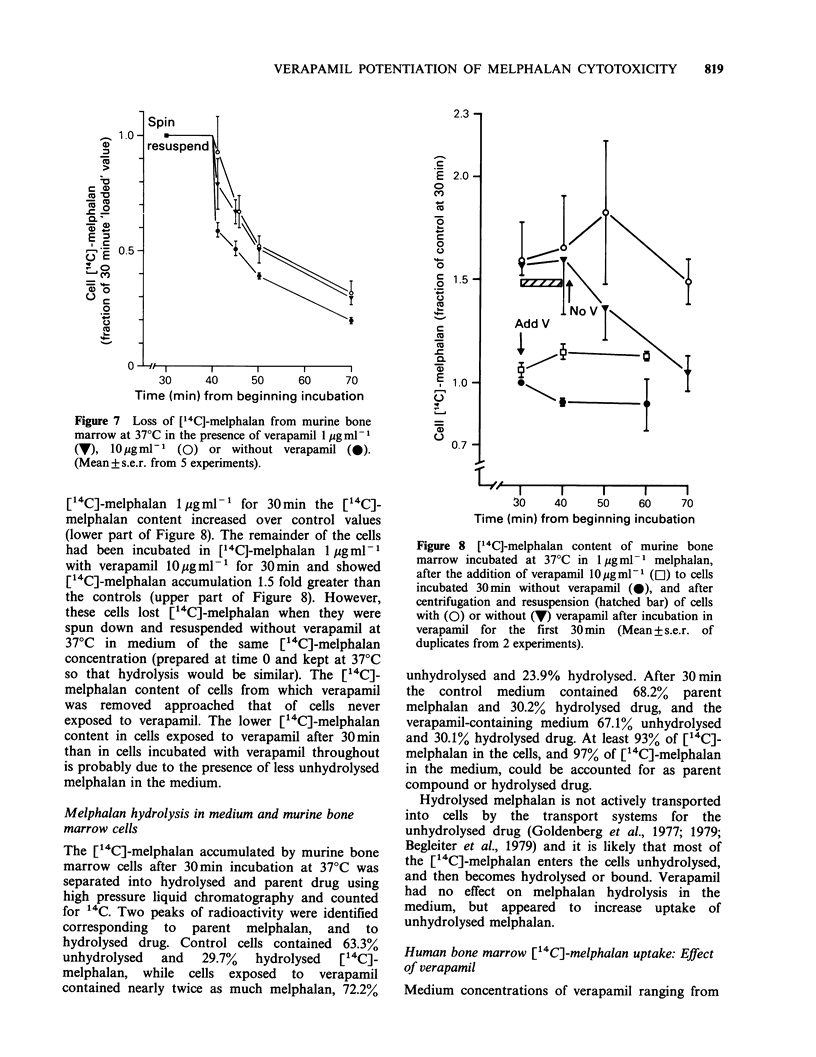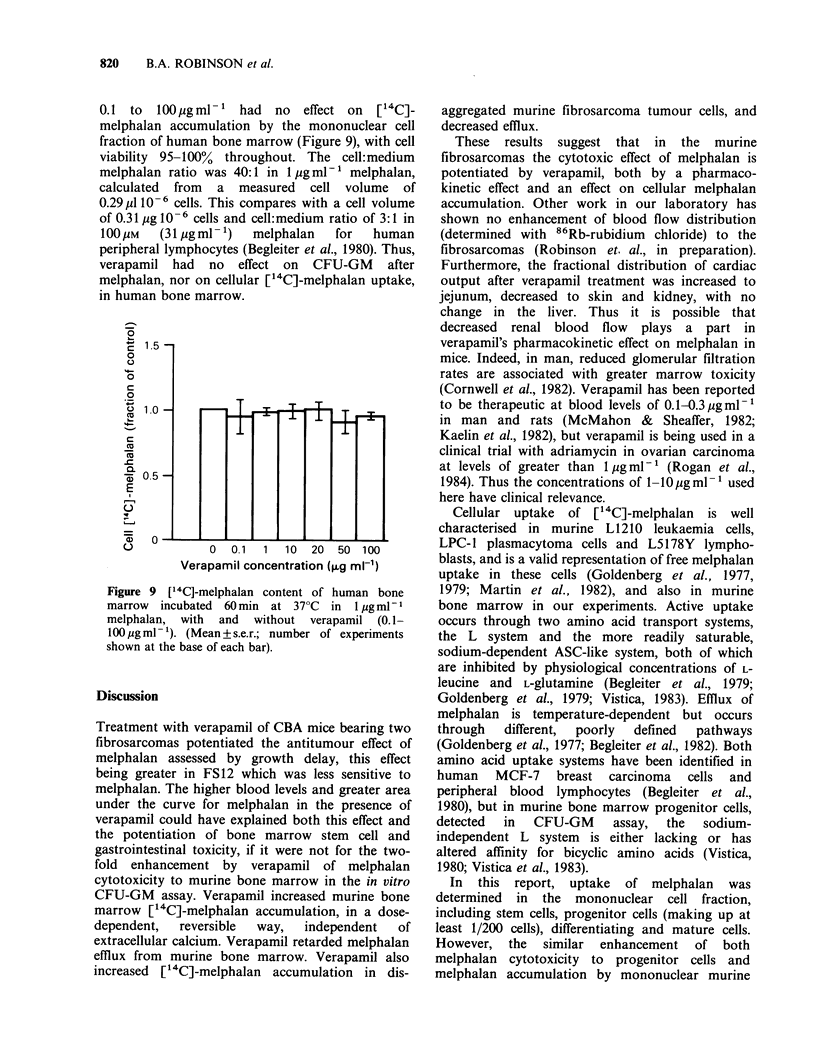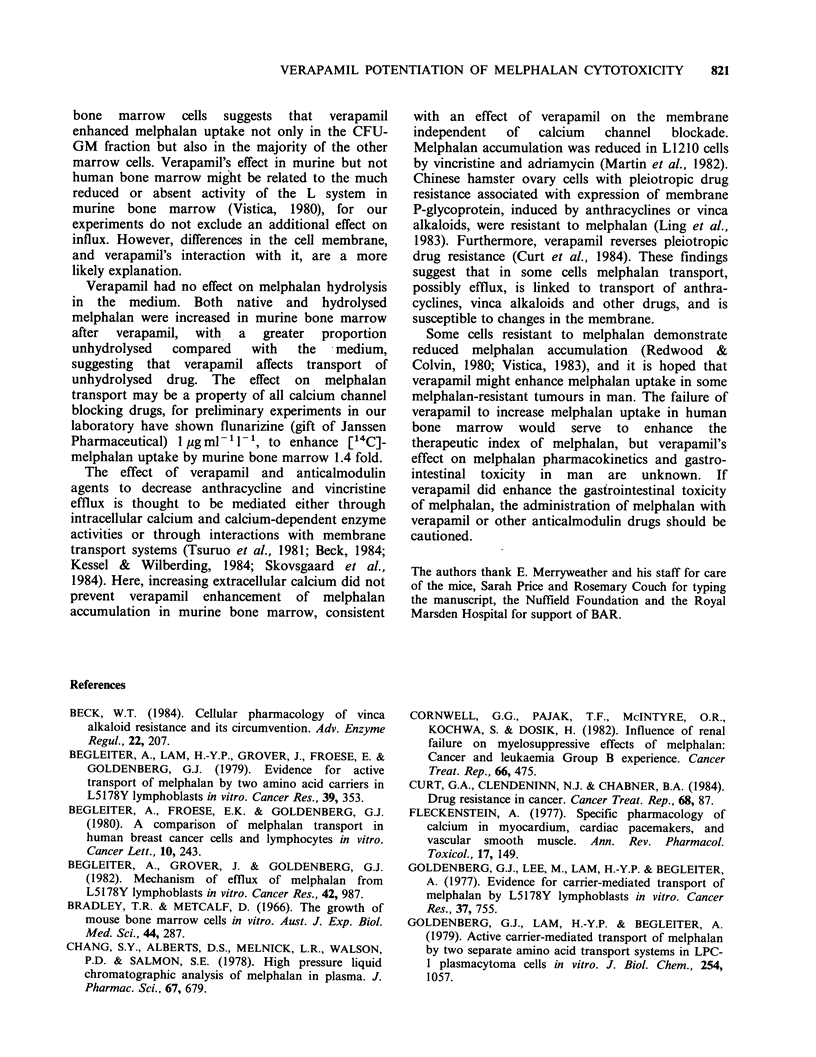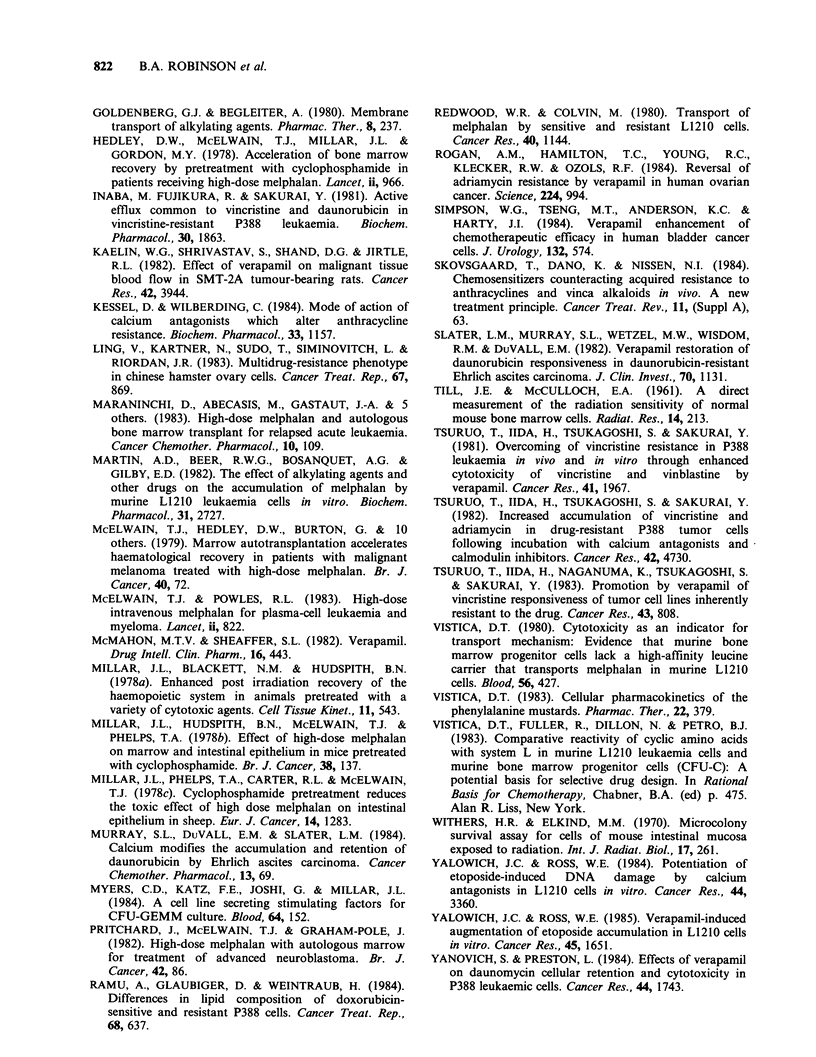# Verapamil potentiation of melphalan cytotoxicity and cellular uptake in murine fibrosarcoma and bone marrow.

**DOI:** 10.1038/bjc.1985.264

**Published:** 1985-12

**Authors:** B. A. Robinson, R. D. Clutterbuck, J. L. Millar, T. J. McElwain

## Abstract

Growth delay by melphalan of two fibrosarcomas in CBA mice was prolonged by intraperitoneal (i.p.) verapamil, 10 mg kg-1. Verapamil also increased the area under the blood concentration time curve and the gastrointestinal toxicity of melphalan. Verapamil promoted melphalan cytotoxicity to murine bone marrow both in vivo, by CFU-S assay, and in vitro, by CFU-GM assay. In 1 microgram ml-1 [14C]-melphalan, verapamil (10 micrograms ml-1) increased by 1.5 times the [14C]-melphalan accumulation by murine bone marrow, reversibly and independently of external calcium. Efflux of [14C]-melphalan from murine bone marrow was retarded by verapamil. Verapamil increased [14C]-melphalan uptake by disaggregated fibrosarcoma cells but had no effect on melphalan accumulation and cytotoxicity in human bone marrow. Although verapamil affected melphalan pharmacokinetics, enhancement of cellular melphalan uptake by verapamil in murine fibrosarcoma and bone marrow appeared to account for much of the increase in melphalan cytotoxicity. The lack of potentiation of melphalan by verapamil in human marrow suggests differences in melphalan transport or in verapamil membrane interactions in mouse and man.


*Br. J. Cancer* (1985), **52**, 813–822

### Verapamil potentiation of melphalan cytotoxicity and cellular uptake in murine fibrosarcoma and bone marrow

B.A. Robinson, R.D. Clutterbuck, J.L. Millar & T.J. McElwain

*Department of Medicine in the Royal Marsden Hospital and Institute of Cancer Research, Sutton, Surrey, UK.*

**Summary** Growth delay by melphalan of two fibrosarcomas in CBA mice was prolonged by intraperitoneal (i.p.) verapamil, $10 \mathrm{mg\,kg^{-1}}$. Verapamil also increased the area under the blood concentration time curve and the gastrointestinal toxicity of melphalan. Verapamil promoted melphalan cytotoxicity to murine bone marrow both *in vivo*, by CFU-S assay, and *in vitro*, by CFU-GM assay. In $1 \mu\mathrm{g\,ml^{-1}}$ [$^{14}$C]-melphalan, verapamil ($10 \mu\mathrm{g\,ml^{-1}}$) increased by 1.5 times the [$^{14}$C]-melphalan accumulation by murine bone marrow, reversibly and independently of external calcium. Efflux of [$^{14}$C]-melphalan from murine bone marrow was retarded by verapamil. Verapamil increased [$^{14}$C]-melphalan uptake by disaggregated fibrosarcoma cells but had no effect on melphalan accumulation and cytotoxicity in human bone marrow. Although verapamil affected melphalan pharmacokinetics, enhancement of cellular melphalan uptake by verapamil in murine fibrosarcoma and bone marrow appeared to account for much of the increase in melphalan cytotoxicity. The lack of potentiation of melphalan by verapamil in human marrow suggests differences in melphalan transport or in verapamil membrane interactions in mouse and man.

Treatment of tumours is limited by their resistance to chemotherapeutic drugs, either inherent or acquired during treatment. Melphalan is widely used in the treatment of multiple myeloma, ovarian and breast cancer, and has recently been used in high intravenous doses in multiple myeloma, acute leukaemia, solid tumours and paediatric tumours (Hedley *et al.*, 1978; Pritchard *et al.*, 1982; Maraninchi *et al.*, 1983; McElwain & Powles, 1983). The dose limiting toxic effects of melphalan are on the bone marrow, which can be circumvented by autologous marrow transplantation (McElwain *et al.*, 1979), and on the gastrointestinal tract (Millar *et al.*, 1978*b,c*). Priming somewhat ameliorates the toxicity of melphalan (Hedley *et al.*, 1978; Millar *et al.*, 1978*a*), but new approaches are needed to improve the therapeutic index and to circumvent resistance.

Verapamil is a calcium channel blocker which reduces excitation-contraction coupling in cardiac and smooth muscle cells (Fleckenstein, 1977). Verapamil increases cellular accumulation of vinca alkaloids and anthracyclines and increases cytotoxicity *in vitro* and *in vivo* in resistant sublines, and some parent lines, of murine P388 leukaemia, Lewis lung carcinoma, Ehrlich ascites, human B16 melanoma, colon adenocarcinoma, bladder and ovarian carcinoma (Tsuruo *et al.*, 1981; 1982; 1983; Rogan *et al.*, 1984; Slater *et al.*, 1982; Simpson *et al.*, 1984; Yanovich & Preston, 1984). Resistance to anthracyclines and vincristine has been associated with increased active efflux of these drugs (Inaba *et al.*, 1981) and with changes in membrane lipid composition (Ramu *et al.*, 1984); verapamil is thought to interact with the cell membrane and decrease active extrusion (Tsuruo *et al.*, 1981; Kessel & Wilberding, 1984; Murray *et al.*, 1984; Skovsgaard *et al.*, 1984).

Melphalan is taken into cells by two separate amino acid transport systems (Goldenberg & Begleiter, 1980; Vistica, 1983), and efflux occurs through different undefined pathways. Rogan *et al.* (1984) reported that verapamil had no effect on the cytotoxicity of melphalan in a melphalan-resistant human ovarian carcinoma cell line. The recent findings of verapamil potentiation of etoposide-induced DNA damage and cytotoxicity in L1210 cells *in vitro*, with decreased etoposide efflux (Yalowich & Ross, 1984; 1985), and the cross resistance to melphalan of chinese hamster ovary cells exhibiting pleiotropic drug resistance (Ling *et al.*, 1983) suggest that drugs other than anthracyclines and vinca alkaloids may be affected by verapamil. We here report enhancement of melphalan-induced growth delay of two murine fibrosarcomas by verapamil, a finding which prompted study of the effects of verapamil on melphalan cytotoxicity and cellular uptake in murine fibrosarcomas and bone marrow, and in human bone marrow, and also effects on pharmacokinetics and normal tissue toxicity in mice.

### Methods

### *Animals and tumours*

Male and female CBA/ca mice, at least 12 weeks

Correspondence: B.A. Robinson.
Received 14 May 1985; and in revised form 5 August 1985.

© The Macmillan Press Ltd., 1985

old were used, and kept at 22°C with free access to food and water. Two fibrosarcomas, FS12 and FS13, obtained from Dr S. Eccles, Institute of Cancer Research (ICR) were passaged every 2–3 weeks as a cell suspension. Tumour was finely minced with crossed scalpels and incubated at 37°C in PBS containing $0.5\,mg\,ml^{-1}$ pronase, $0.2\,mg\,ml^{-1}$ DNAse and $0.2\,mg\,ml^{-1}$ collagenase. The disaggregated tumour cells were washed in PBS and adjusted to $7\times10^6$ cells $ml^{-1}$. Female CBA mice, 12–24 weeks old, average weight 20 g (range 16–24 g) were given 0.1 ml cell suspension by s.c. injection into each flank. Experiments were performed on passages 12–15 of FS12 and 5–12 of FS13.

*Media and drugs*

Balanced salt solution (BSS) and PBS were obtained from the central sterile supplies department, ICR. PBS and BSS contained no calcium and no magnesium. Human and mouse alpha media with 20% newborn calf serum were used in clonogenic assays.

Melphalan (Alkeran, Burrough's Wellcome, UK) was dissolved in acid alcohol (5 M HCl:ethanol 1:50), $20\,mg\,ml^{-1}$, stored below 0°C, and diluted in saline immediately before injection. For cell uptake studies [$^{14}$C]-melphalan labelled in the chloroethyl side chain, specific activity $33.7\,\mu Ci\,mg^{-1}$ (SRI International), was dissolved in acid alcohol, $1\,mg\,ml^{-1}$, and diluted in PBS to $1\,\mu g\,ml^{-1}$ ($3.2\,\mu M$) at the start of incubation. For pharmacokinetic studies, melphalan in acid alcohol was 'spiked' with [$^{14}$C]-melphalan. Verapamil hydrochloride (Cordilox, Abbott) was diluted in saline.

*Growth delay*

Female CBA mice bearing bilateral s.c. fibrosarcomas were divided into groups of 5 mice with tumours of the same size distribution. On day 0 (2 weeks after transplant), mice were treated with verapamil $10\,mg\,kg^{-1}$ i.p., melphalan $10\,mg\,kg^{-1}$ i.p. or both drugs simultaneously, and a fourth group were untreated. Tumour volume was calculated from $V=\pi Dd^2/6$ where $D$ is the longest diameter and $d$ the perpendicular diameter. Volume at various times ($Vt$) was expressed as a ratio of the volume on day 0 ($Vo$). The mean ± s.e. of the ratio (s.e.r.) was calculated for each group.

*Pharmacokinetics of [$^{14}$C]-melphalan in mice*

The blood levels of [$^{14}$C]-melphalan after $10\,mg\,kg^{-1}$ melphalan i.p. were determined in groups of 3 CBA mice in the presence and absence of simultaneous i.p. verapamil in doses of 2.5, 5 and $10\,mg\,kg^{-1}$. Tail vein blood ($20\,\mu l$) samples taken over 4 h were assayed for $^{14}$C, using a Packard Oxidiser 306 (United Technologies Packard). In an automatic sequence, $^{14}$C was trapped in 9 ml of Carbosorb (Packard) and added to 13 ml of Permafluor V scintillant (Packard). Samples and dose standards were counted in a liquid scintillation counter, and [$^{14}$C]-melphalan expressed as a percentage of the dose per ml of blood. Means ± s.e. were compared using the Student's $t$ test.

*Jejunum microcolony assay*

Groups of 3 or 4 CBA mice were treated with melphalan i.p. and/or verapamil $10\,mg\,kg^{-1}$ i.p. and killed 4 days later. Three portions of jejunum per mouse were excised, fixed and 4–5 $\mu m$ thick transverse sections stained with H&E. Surviving cryptogenic cells were assessed using a technique modified from Withers and Elkind (1970) (Millar *et al.*, 1978*b*). The number of regenerating crypts per circumference was expressed as a fraction of the number of crypts per circumference in untreated normal mice, and compared as the mean ± s.e.r. using the $t$ test for small samples.

*Spleen colony assay*

The spleen colony forming cells of mouse bone marrow (CFU-S) were assayed by the method of Till and McCulloch (1961). Mice were treated with i.p. melphalan and/or verapamil and were killed with control animals one day later for CFU-S assay. Results of 3 experiments were similar and were combined.

*Clonogenic assay, CFU-GM*

The mononuclear fraction of human and mouse bone marrow was obtained by centrifugation at 400 *g* for 30 min over a Ficoll/sodium metrizoate solution (Lymphoprep, specific gravity 1.077, Nyegaard). Cells were incubated in the presence of melphalan ($0.01–2\,\mu g\,ml^{-1}$) and/or verapamil $10\,\mu g\,ml^{-1}$ at 37°C for 60 min, washed and resuspended in drug-free medium. Then the colony forming ability of the mononuclear cell fraction (CFU-GM) was assayed following the method of Bradley and Metcalf (1966) plating each treatment group in triplicate. Colony stimulating factor was derived from pregnant mouse uterus (PMUE) for mouse marrow, and from 5637 human bladder carcinoma cells for human marrow (Myers *et al.*, 1984).

In vitro *[$^{14}$C]-melphalan uptake and efflux*

The mononuclear cell fraction of bone marrow and disaggregated fibrosarcoma cells, separated from red cells by centrifugation over Lymphoprep, were adjusted to 2–3 × $10^6$ cells $ml^{-1}$ in PBS. No amino acids were present in the medium (Goldenberg & Begleiter, 1980; Vistica, 1983). Verapamil (0.1 to 100 $\mu g\,ml^{-1}$) was added, and finally [$^{14}C$]-melphalan, 1 $\mu g\,ml^{-1}$, and the cells incubated at 37°C for 60 min. Then using a method adapted from Martin *et al.* (1982), duplicate 1 ml aliquots were layered over 0.5 ml oil (mineral oil:silicone oil (Dow Corning), 1:4) and spun at 12,000 *g* for 2–3 min in a microcentrifuge 320a (Burkard Scientific Sales Ltd). The cell pellets were solubilised in Protosol (New England Nuclear) and added to 10 ml of ACS scintillant (New England Nuclear) containing acetic acid 37.5 ml $gallon^{-1}$. Samples and aliquots of supernatant were counted in a liquid scintillation counter (Intertechnique). Cell $^{14}C$ was calculated as ng [$^{14}C$]-melphalan per $10^6$ cells, and then expressed as a fraction of [$^{14}C$]-melphalan in control cells after 60 min in verapamil-free, calcium-free medium. No correction was made for membrane binding of [$^{14}C$]-melphalan, usually <5% of the steady state [$^{14}C$]-melphalan level (Goldenberg *et al.*, 1979; Martin *et al.*, 1982), because plateau levels of [$^{14}C$]-melphalan were compared. The results from 2 or more experiments were combined, as mean ± s.e.r.

For [$^{14}C$]-melphalan efflux studies, cells were loaded by 30 min incubation in 1 $\mu g\,ml^{-1}$ [$^{14}C$]-melphalan. Duplicate 1 ml samples were taken (and spun through oil) and the remaining cells spun down and resuspended in ice cold medium to prevent immediate efflux (Goldenberg *et al.*, 1977; Begleiter *et al.*, 1982; Martin *et al.*, 1982). The cells were then incubated at 37°C without melphalan, in the presence or absence of verapamil, with samples being taken immediately and up to 30 min later.

In one experiment, murine bone marrow cells were incubated 30 min at 37°C with [$^{14}C$]-melphalan, with or without verapamil. Immediately after spinning through oil, samples of supernatant medium and the cell pellets were frozen to stop melphalan hydrolysis. The cells were ultrasonicated in 100% methanol and 50 $\mu l$ samples immediately analysed by high pressure liquid chromatography (Laboratory Data Control), at absorbance 263 nm, 0.001 AUFS sensitivity, using 1:1 methanol:water with 1% acetic acid solvent (Fisons, HPLC grade) and 1.4 ml $min^{-1}$ flow rate, in a method adapted from Chang *et al.* (1978). The effluent was collected in 30 second fractions and counted for $^{14}C$, in 10 ml of ACS scintillant. The medium was thawed, immediately diluted with methanol and analysed similarly. Verapamil had no effect on the rate of hydrolysis of melphalan in PBS at 37°C.

At the beginning and end of each experiment, and following resuspension, cells were counted using a Coulter Counter (Coulter Electronics) and viability determined by trypan blue dye exclusion. Cell size was determined using a modified Coulter Counter calibrated with latex particles.

### Results

### *Growth delay of murine fibrosarcomas by melphalan: Effect of verapamil*

The doubling time of FS12 was 5 days. The growth delay by melphalan 10 mg $kg^{-1}$ i.p. was increased from 6 days to 16 days when verapamil 10 mg $kg^{-1}$ i.p. was also administered (Figure 1). One tumour from the melphalan group and three from the melphalan with verapamil group regressed completely, so the real difference may be greater. For FS13, verapamil increased the growth delay by melphalan 10 mg $kg^{-1}$ i.p. from 15 days to 22 days (Data not shown). The fibrosarcoma less sensitive to melphalan, FS12, showed greater enhancement of growth delay with verapamil.

**Figure 1** Growth delay of FS12 fibrosarcoma in CBA mice treated with melphalan 10 mg $kg^{-1}$ i.p. (○) $n = 9$; verapamil 10 mg $kg^{-1}$ i.p. (□) $n = 8$; both (■) $n = 8$; or untreated (●) $n = 9$. (Mean ± s.e.r. of 8–9 tumours; Vt/Vo is tumour volume as a fraction of volume on day 0).

### *Pharmacokinetics of [$^{14}C$]-melphalan in mice: Effect of verapamil*

Administration of verapamil 10 mg $kg^{-1}$ i.p. with melphalan 10 mg $kg^{-1}$ i.p. significantly increased blood [$^{14}C$]-melphalan levels and the area under the curve (Figure 2). A similar increase in blood [$^{14}C$]-melphalan levels was seen when melphalan was given i.v. with verapamil 10 mg $kg^{-1}$ i.p. (result not shown), excluding the possibility that verapamil

**Figure 2** Blood levels of [$^{14}$C]-melphalan in CBA mice treated with [$^{14}$C]-melphalan 10 mg kg$^{-1}$ i.p. alone (●) or with i.p. verapamil (▲). (Mean ± s.e. of 3 mice).

**Figure 3** Jejunum microcolonies in CBA mice after i.p. melphalan, alone (●) or with verapamil 10 mg kg$^{-1}$ i.p. (○). (Mean ± s.e.r. of 3–4 mice, 2 separate experiments).

increased absorption of i.p. melphalan. This pharmacokinetic effect was still present with verapamil 5 mg kg$^{-1}$ i.p. but was negligible for verapamil 2.5 mg kg$^{-1}$ i.p. (Figure 2).

*Normal tissue toxicity of melphalan in mice: Effect of verapamil*

The jejunum microcolony assay, performed twice, showed potentiation of the toxicity of melphalan by 10 mg kg$^{-1}$ verapamil i.p. for melphalan doses of 15–20 mg kg$^{-1}$ i.p. (Figure 3). In the CFU-S assay melphalan cytotoxicity to murine bone marrow stem cells was increased significantly by treatment with verapamil 10 mg kg$^{-1}$ i.p. but not by the lower doses of 5 and 2.5 mg kg$^{-1}$ i.p. (Figure 4). Verapamil 10 mg kg$^{-1}$ i.p. alone had no effect in either assay. Thus verapamil increased melphalan toxicity to the two most sensitive normal tissues, gastrointestinal tract and bone marrow.

**Figure 4** Spleen colonies (CFU-S) in CBA mice after treatment with i.p. melphalan, alone (○), or with i.p. verapamil, 2.5 mg kg$^{-1}$ (▲), 5 mg kg$^{-1}$ (■) or 10 mg kg$^{-1}$ (●). (Mean ± s.e.r., pooled from 3 experiments).

### *Murine and human bone marrow CFU-GM with melphalan: Effect of verapamil*

The potentiation by verapamil of melphalan-induced growth delay and of melphalan toxicity could be due to the pharmacokinetic effect. However, the following *in vitro* experiments suggested that verapamil had a direct effect on murine bone marrow. When the mononuclear cell fraction of murine bone marrow was exposed to melphalan in the presence and absence of verapamil for 60 min, verapamil 10 $\mu g\,ml^{-1}$ enhanced melphalan cytotoxicity, approximately two-fold (Figure 5a). Plating efficiency with PMUE was 1:150–1:200. Verapamil 10 $\mu g\,ml^{-1}$ had a slight cytotoxic effect on murine bone marrow which compares with minimal cytotoxicity of verapamil at 23 $\mu M$ (10 $\mu g\,ml^{-1}$) for P388 cells and at 6.6 $\mu M$ (3 $\mu g\,ml^{-1}$) for 4 tumour cell lines (Tsuruo *et al.*, 1981; 1983).

In contrast, verapamil (10 $\mu g\,ml^{-1}$) had no effect on melphalan cytotoxicity to the mononuclear cell fraction of human bone marrow (Figure 5b). The plating efficiency of human marrow was 1:700.

### *Cellular [$^{14}C$]-melphalan uptake by murine bone marrow and fibrosarcoma: Effect of verapamil*

Verapamil enhancement of *in vitro* melphalan cytotoxicity raised the possibility of an effect on melphalan uptake, and this was explored in detail. Accumulation of [$^{14}C$]-melphalan by the mononuclear cell fraction of mouse bone marrow after 60 min incubation was greater in the presence of verapamil (Figure 6) with a 1.5 fold enhancement at a verapamil concentration of 10 $\mu g\,ml^{-1}$. Disaggregated fibrosarcoma cells also accumulated more [$^{14}C$]-melphalan in the presence of verapamil 10 $\mu g\,ml^{-1}$ with a greater effect for FS12 (Figure 6). Fibrosarcoma FS13 cells, disaggregated *without* incubation in enzymes, also took up 1.22 fold more [$^{14}C$]-melphalan in the presence of verapamil 10 $\mu g\,ml^{-1}$, in 2 experiments, making it unlikely that the effect was due to enzymic damage to the cell membrane.

Uptake of [$^{14}C$]-melphalan was initially rapid, and reached a plateau after 10–20 min for both murine marrow and fibrosarcoma cells as occurs in murine L1210 leukaemia cells, L5178Y lymphoblasts and LPC-1 plasmacytoma cells (Goldenberg *et al.*, 1977; Martin *et al.*, 1982; Vistica, 1983). For murine bone marrow, cellular [$^{14}C$]-melphalan was already 1.4 fold greater in verapamil treated cells than in controls after 10 min (Table I). The cell volume of mononuclear mouse bone marrow cells was 0.35 $\mu l\,10^{-6}$ cells and the cell:medium melphalan ratio was 15:1. The viability of murine bone marrow cells was 90–100% and of fibrosarcoma cells 70–85%, and remained constant within each experiment during the 60 min incubation.

Verapamil's calcium channel blocking action is overcome by increasing extracellular calcium concentration (Fleckenstein, 1977). However,

**Figure 5** CFU-GM for murine bone marrow (a) and human bone marrow (b) after incubation at 37°C for 60 min with melphalan, alone (●) or with verapamil 10 $\mu g\,ml^{-1}$ (○). Mean ± s.e.r. from 5(a) and 7(b) experiments.

**Figure 6** [$^{14}$C]-melphalan content of (a) mouse bone marrow and (b) disaggregated murine fibrosarcoma cells, incubated 60 min at 37°C in 1 $\mu$g ml$^{-1}$ [$^{14}$C]-melphalan, with and without verapamil (0.1–100 $\mu$g ml$^{-1}$). (Mean ± s.e.r.; number of experiments shown in the base of each bar).

addition of calcium to the medium had no effect on [$^{14}$C]-melphalan uptake, nor on its enhancement by verapamil 10 $\mu$g ml$^{-1}$, in murine bone marrow (Table II). Because high concentrations of calcium precipitated phosphate when added to PBS, the second experiment (b) was performed in BSS. Although addition of melphalan (in acid alcohol) to BSS lowered pH cell viability was maintained for the 60 min.

**Table I** Accumulation of [$^{14}$C]-melphalan by the mononuclear cell fraction of murine bone marrow, incubated with 1 $\mu$g ml$^{-1}$ [$^{14}$C]-melphalan at 37°C, with or without verapamil 10 $\mu$g ml$^{-1}$

| *Duration of incubation (min)* | *Cellular [$^{14}$C]-melphalan ratio: verapamil/control* | *No. of experiments* |
|---|---|---|
| 10 | 1.41 ± 0.10 | 2 |
| 30 | 1.45 ± 0.08 | 4 |
| 60 | 1.47 ± 0.15 | 9 |

(mean ± s.e.r.).

**Table II** [$^{14}$C]-melphalan accumulation by murine bone marrow after 60 min in 1 $\mu$g ml$^{-1}$ melphalan, ± verapamil 10 $\mu$g ml$^{-1}$, at 37°C with or without calcium.

| | | | |
|---|---|---|---|
| (a) Medium Ca (mM) (in PBS) | 0 | 0.125 | 0.25 |
| Control | 1.00 ± 0.07 | 1.12 ± 0.07 | 1.15 ± 0.05 |
| Verapamil | 1.50 ± 0.18 | 1.53 ± 0.10 | 1.47 ± 0.08 |
| (b) Medium Ca (mM) (in BSS) | 0 | 1.25 | 2.50 |
| Control | 1.00 ± 0.06 | 1.02 ± 0.13 | 1.07 ± 0.06 |
| Verapamil | 1.18 ± 0.06 | 1.46 ± 0.10 | 1.34 ± 0.06 |

Uptake as fraction of control without calcium. Mean ± s.e.r. of duplicates (a) or triplicates (b).

*Efflux of [$^{14}$C]-melphalan from murine bone marrow: Effect of verapamil*

Verapamil appears to decrease extrusion of anthracyclines and vinca alkaloids from resistant cells (Tsuruo *et al.*, 1981; Skovsgaard *et al.*, 1984) so the effect of verapamil on melphalan efflux was studied. When the mononuclear cell fraction of murine bone marrow, loaded by incubation in [$^{14}$C]-melphalan 1 $\mu$g ml$^{-1}$ for 30 min, was resuspended in melphalan-free medium, the presence of verapamil (1 and 10 $\mu$g ml$^{-1}$) retarded loss of [$^{14}$C]-melphalan from the cells (Figure 7). Cell viability was always 90–100%.

This experiment was repeated, using disaggregated murine fibrosarcoma FS13 cells, loaded with [$^{14}$C]-melphalan by 30 min incubation at 37°C in 1 $\mu$g ml$^{-1}$. Thirty minutes after resuspension in melphalan-free medium, with or without verapamil 10 $\mu$g ml$^{-1}$, cells exposed to verapamil retained 47% of the [$^{14}$C]-melphalan present at 30 min, compared with 36% for control cells. Thus it appears likely that verapamil decreases efflux of [$^{14}$C]-melphalan from the fibrosarcoma cells as well.

The following experiment using murine bone marrow was performed twice, each time with duplicate samples. When verapamil 10 $\mu$g ml$^{-1}$ was added to cells which had already been incubated in

**Figure 7** Loss of [$^{14}$C]-melphalan from murine bone marrow at 37°C in the presence of verapamil 1 $\mu$g ml$^{-1}$ (▼), 10 $\mu$g ml$^{-1}$ (○) or without verapamil (●). (Mean ± s.e.r. from 5 experiments).

[$^{14}$C]-melphalan 1 $\mu$g ml$^{-1}$ for 30 min the [$^{14}$C]-melphalan content increased over control values (lower part of Figure 8). The remainder of the cells had been incubated in [$^{14}$C]-melphalan 1 $\mu$g ml$^{-1}$ with verapamil 10 $\mu$g ml$^{-1}$ for 30 min and showed [$^{14}$C]-melphalan accumulation 1.5 fold greater than the controls (upper part of Figure 8). However, these cells lost [$^{14}$C]-melphalan when they were spun down and resuspended without verapamil at 37°C in medium of the same [$^{14}$C]-melphalan concentration (prepared at time 0 and kept at 37°C so that hydrolysis would be similar). The [$^{14}$C]-melphalan content of cells from which verapamil was removed approached that of cells never exposed to verapamil. The lower [$^{14}$C]-melphalan content in cells exposed to verapamil after 30 min than in cells incubated with verapamil throughout is probably due to the presence of less unhydrolysed melphalan in the medium.

*Melphalan hydrolysis in medium and murine bone marrow cells*

The [$^{14}$C]-melphalan accumulated by murine bone marrow cells after 30 min incubation at 37°C was separated into hydrolysed and parent drug using high pressure liquid chromatography and counted for $^{14}$C. Two peaks of radioactivity were identified corresponding to parent melphalan, and to hydrolysed drug. Control cells contained 63.3% unhydrolysed and 29.7% hydrolysed [$^{14}$C]-melphalan, while cells exposed to verapamil contained nearly twice as much melphalan, 72.2%

**Figure 8** [$^{14}$C]-melphalan content of murine bone marrow incubated at 37°C in 1 $\mu$g ml$^{-1}$ melphalan, after the addition of verapamil 10 $\mu$g ml$^{-1}$ (□) to cells incubated 30 min without verapamil (●), and after centrifugation and resuspension (hatched bar) of cells with (○) or without (▼) verapamil after incubation in verapamil for the first 30 min (Mean ± s.e.r. of duplicates from 2 experiments).

unhydrolysed and 23.9% hydrolysed. After 30 min the control medium contained 68.2% parent melphalan and 30.2% hydrolysed drug, and the verapamil-containing medium 67.1% unhydrolysed and 30.1% hydrolysed drug. At least 93% of [$^{14}$C]-melphalan in the cells, and 97% of [$^{14}$C]-melphalan in the medium, could be accounted for as parent compound or hydrolysed drug.

Hydrolysed melphalan is not actively transported into cells by the transport systems for the unhydrolysed drug (Goldenberg *et al.*, 1977; 1979; Begleiter *et al.*, 1979) and it is likely that most of the [$^{14}$C]-melphalan enters the cells unhydrolysed, and then becomes hydrolysed or bound. Verapamil had no effect on melphalan hydrolysis in the medium, but appeared to increase uptake of unhydrolysed melphalan.

*Human bone marrow [$^{14}$C]-melphalan uptake: Effect of verapamil*

Medium concentrations of verapamil ranging from

0.1 to 100 $\mu$g ml$^{-1}$ had no effect on [$^{14}$C]-melphalan accumulation by the mononuclear cell fraction of human bone marrow (Figure 9), with cell viability 95–100% throughout. The cell:medium melphalan ratio was 40:1 in 1 $\mu$g ml$^{-1}$ melphalan, calculated from a measured cell volume of 0.29 $\mu$l 10$^{-6}$ cells. This compares with a cell volume of 0.31 $\mu$g 10$^{-6}$ cells and cell:medium ratio of 3:1 in 100 $\mu$M (31 $\mu$g ml$^{-1}$) melphalan for human peripheral lymphocytes (Begleiter *et al.*, 1980). Thus, verapamil had no effect on CFU-GM after melphalan, nor on cellular [$^{14}$C]-melphalan uptake, in human bone marrow.

**Figure 9** [$^{14}$C]-melphalan content of human bone marrow incubated 60 min at 37°C in 1 $\mu$g ml$^{-1}$ melphalan, with and without verapamil (0.1–100 $\mu$g ml$^{-1}$). (Mean $\pm$ s.e.r.; number of experiments shown at the base of each bar).

### Discussion

Treatment with verapamil of CBA mice bearing two fibrosarcomas potentiated the antitumour effect of melphalan assessed by growth delay, this effect being greater in FS12 which was less sensitive to melphalan. The higher blood levels and greater area under the curve for melphalan in the presence of verapamil could have explained both this effect and the potentiation of bone marrow stem cell and gastrointestinal toxicity, if it were not for the two-fold enhancement by verapamil of melphalan cytotoxicity to murine bone marrow in the *in vitro* CFU-GM assay. Verapamil increased murine bone marrow [$^{14}$C]-melphalan accumulation, in a dose-dependent, reversible way, independent of extracellular calcium. Verapamil retarded melphalan efflux from murine bone marrow. Verapamil also increased [$^{14}$C]-melphalan accumulation in disaggregated murine fibrosarcoma tumour cells, and decreased efflux.

These results suggest that in the murine fibrosarcomas the cytotoxic effect of melphalan is potentiated by verapamil, both by a pharmacokinetic effect and an effect on cellular melphalan accumulation. Other work in our laboratory has shown no enhancement of blood flow distribution (determined with $^{86}$Rb-rubidium chloride) to the fibrosarcomas (Robinson *et. al.*, in preparation). Furthermore, the fractional distribution of cardiac output after verapamil treatment was increased to jejunum, decreased to skin and kidney, with no change in the liver. Thus it is possible that decreased renal blood flow plays a part in verapamil's pharmacokinetic effect on melphalan in mice. Indeed, in man, reduced glomerular filtration rates are associated with greater marrow toxicity (Cornwell *et al.*, 1982). Verapamil has been reported to be therapeutic at blood levels of 0.1–0.3 $\mu$g ml$^{-1}$ in man and rats (McMahon & Sheaffer, 1982; Kaelin *et al.*, 1982), but verapamil is being used in a clinical trial with adriamycin in ovarian carcinoma at levels of greater than 1 $\mu$g ml$^{-1}$ (Rogan *et al.*, 1984). Thus the concentrations of 1–10 $\mu$g ml$^{-1}$ used here have clinical relevance.

Cellular uptake of [$^{14}$C]-melphalan is well characterised in murine L1210 leukaemia cells, LPC-1 plasmacytoma cells and L5178Y lymphoblasts, and is a valid representation of free melphalan uptake in these cells (Goldenberg *et al.*, 1977, 1979; Martin *et al.*, 1982), and also in murine bone marrow in our experiments. Active uptake occurs through two amino acid transport systems, the L system and the more readily saturable, sodium-dependent ASC-like system, both of which are inhibited by physiological concentrations of L-leucine and L-glutamine (Begleiter *et al.*, 1979; Goldenberg *et al.*, 1979; Vistica, 1983). Efflux of melphalan is temperature-dependent but occurs through different, poorly defined pathways (Goldenberg *et al.*, 1977; Begleiter *et al.*, 1982). Both amino acid uptake systems have been identified in human MCF-7 breast carcinoma cells and peripheral blood lymphocytes (Begleiter *et al.*, 1980), but in murine bone marrow progenitor cells, detected in CFU-GM assay, the sodium-independent L system is either lacking or has altered affinity for bicyclic amino acids (Vistica, 1980; Vistica *et al.*, 1983).

In this report, uptake of melphalan was determined in the mononuclear cell fraction, including stem cells, progenitor cells (making up at least 1/200 cells), differentiating and mature cells. However, the similar enhancement of both melphalan cytotoxicity to progenitor cells and melphalan accumulation by mononuclear murine bone marrow cells suggests that verapamil enhanced melphalan uptake not only in the CFU-GM fraction but also in the majority of the other marrow cells. Verapamil's effect in murine but not human bone marrow might be related to the much reduced or absent activity of the L system in murine bone marrow (Vistica, 1980), for our experiments do not exclude an additional effect on influx. However, differences in the cell membrane, and verapamil's interaction with it, are a more likely explanation.

Verapamil had no effect on melphalan hydrolysis in the medium. Both native and hydrolysed melphalan were increased in murine bone marrow after verapamil, with a greater proportion unhydrolysed compared with the medium, suggesting that verapamil affects transport of unhydrolysed drug. The effect on melphalan transport may be a property of all calcium channel blocking drugs, for preliminary experiments in our laboratory have shown flunarizine (gift of Janssen Pharmaceutical) $1 \mu g\,ml^{-1}\,l^{-1}$, to enhance [$^{14}$C]-melphalan uptake by murine bone marrow 1.4 fold.

The effect of verapamil and anticalmodulin agents to decrease anthracycline and vincristine efflux is thought to be mediated either through intracellular calcium and calcium-dependent enzyme activities or through interactions with membrane transport systems (Tsuruo *et al.*, 1981; Beck, 1984; Kessel & Wilberding, 1984; Skovsgaard *et al.*, 1984). Here, increasing extracellular calcium did not prevent verapamil enhancement of melphalan accumulation in murine bone marrow, consistent with an effect of verapamil on the membrane independent of calcium channel blockade. Melphalan accumulation was reduced in L1210 cells by vincristine and adriamycin (Martin *et al.*, 1982). Chinese hamster ovary cells with pleiotropic drug resistance associated with expression of membrane P-glycoprotein, induced by anthracyclines or vinca alkaloids, were resistant to melphalan (Ling *et al.*, 1983). Furthermore, verapamil reverses pleiotropic drug resistance (Curt *et al.*, 1984). These findings suggest that in some cells melphalan transport, possibly efflux, is linked to transport of anthracyclines, vinca alkaloids and other drugs, and is susceptible to changes in the membrane.

Some cells resistant to melphalan demonstrate reduced melphalan accumulation (Redwood & Colvin, 1980; Vistica, 1983), and it is hoped that verapamil might enhance melphalan uptake in some melphalan-resistant tumours in man. The failure of verapamil to increase melphalan uptake in human bone marrow would serve to enhance the therapeutic index of melphalan, but verapamil's effect on melphalan pharmacokinetics and gastrointestinal toxicity in man are unknown. If verapamil did enhance the gastrointestinal toxicity of melphalan, the administration of melphalan with verapamil or other anticalmodulin drugs should be cautioned.

The authors thank E. Merryweather and his staff for care of the mice, Sarah Price and Rosemary Couch for typing the manuscript, the Nuffield Foundation and the Royal Marsden Hospital for support of BAR.

### References

BECK, W.T. (1984). Cellular pharmacology of vinca alkaloid resistance and its circumvention. *Adv. Enzyme Regul.*, **22**, 207.

BEGLEITER, A., LAM, H.-Y.P., GROVER, J., FROESE, E. & GOLDENBERG, G.J. (1979). Evidence for active transport of melphalan by two amino acid carriers in L5178Y lymphoblasts *in vitro*. *Cancer Res.*, **39**, 353.

BEGLEITER, A., FROESE, E.K. & GOLDENBERG, G.J. (1980). A comparison of melphalan transport in human breast cancer cells and lymphocytes *in vitro*. *Cancer Lett.*, **10**, 243.

BEGLEITER, A., GROVER, J. & GOLDENBERG, G.J. (1982). Mechanism of efflux of melphalan from L5178Y lymphoblasts *in vitro*. *Cancer Res.*, **42**, 987.

BRADLEY, T.R. & METCALF, D. (1966). The growth of mouse bone marrow cells *in vitro*. *Aust. J. Exp. Biol. Med. Sci.*, **44**, 287.

CHANG, S.Y., ALBERTS, D.S., MELNICK, L.R., WALSON, P.D. & SALMON, S.E. (1978). High pressure liquid chromatographic analysis of melphalan in plasma. *J. Pharmac. Sci.*, **67**, 679.

CORNWELL, G.G., PAJAK, T.F., McINTYRE, O.R., KOCHWA, S. & DOSIK, H. (1982). Influence of renal failure on myelosuppressive effects of melphalan: Cancer and leukaemia Group B experience. *Cancer Treat. Rep.*, **66**, 475.

CURT, G.A., CLENDENINN, N.J. & CHABNER, B.A. (1984). Drug resistance in cancer. *Cancer Treat. Rep.*, **68**, 87.

FLECKENSTEIN, A. (1977). Specific pharmacology of calcium in myocardium, cardiac pacemakers, and vascular smooth muscle. *Ann. Rev. Pharmacol. Toxicol.*, **17**, 149.

GOLDENBERG, G.J., LEE, M., LAM, H.-Y.P. & BEGLEITER, A. (1977). Evidence for carrier-mediated transport of melphalan by L5178Y lymphoblasts *in vitro*. *Cancer Res.*, **37**, 755.

GOLDENBERG, G.J., LAM, H.-Y.P. & BEGLEITER, A. (1979). Active carrier-mediated transport of melphalan by two separate amino acid transport systems in LPC-1 plasmacytoma cells *in vitro*. *J. Biol. Chem.*, **254**, 1057.

GOLDENBERG, G.J. & BEGLEITER, A. (1980). Membrane transport of alkylating agents. *Pharmac. Ther.*, **8**, 237.

HEDLEY, D.W., McELWAIN, T.J., MILLAR, J.L. & GORDON, M.Y. (1978). Acceleration of bone marrow recovery by pretreatment with cyclophosphamide in patients receiving high-dose melphalan. *Lancet*, **ii**, 966.

INABA, M. FUJIKURA, R. & SAKURAI, Y. (1981). Active efflux common to vincristine and daunorubicin in vincristine-resistant P388 leukaemia. *Biochem. Pharmacol.*, **30**, 1863.

KAELIN, W.G., SHRIVASTAV, S., SHAND, D.G. & JIRTLE, R.L. (1982). Effect of verapamil on malignant tissue blood flow in SMT-2A tumour-bearing rats. *Cancer Res.*, **42**, 3944.

KESSEL, D. & WILBERDING, C. (1984). Mode of action of calcium antagonists which alter anthracycline resistance. *Biochem. Pharmacol.*, **33**, 1157.

LING, V., KARTNER, N., SUDO, T., SIMINOVITCH, L. & RIORDAN, J.R. (1983). Multidrug-resistance phenotype in chinese hamster ovary cells. *Cancer Treat. Rep.*, **67**, 869.

MARANINCHI, D., ABECASIS, M., GASTAUT, J.-A. & 5 others. (1983). High-dose melphalan and autologous bone marrow transplant for relapsed acute leukaemia. *Cancer Chemother. Pharmacol.*, **10**, 109.

MARTIN, A.D., BEER, R.W.G., BOSANQUET, A.G. & GILBY, E.D. (1982). The effect of alkylating agents and other drugs on the accumulation of melphalan by murine L1210 leukaemia cells *in vitro*. *Biochem. Pharmacol.*, **31**, 2727.

McELWAIN, T.J., HEDLEY, D.W., BURTON, G. & 10 others. (1979). Marrow autotransplantation accelerates haematological recovery in patients with malignant melanoma treated with high-dose melphalan. *Br. J. Cancer*, **40**, 72.

McELWAIN, T.J. & POWLES, R.L. (1983). High-dose intravenous melphalan for plasma-cell leukaemia and myeloma. *Lancet*, **ii**, 822.

McMAHON, M.T.V. & SHEAFFER, S.L. (1982). Verapamil. *Drug Intell. Clin. Pharm.*, **16**, 443.

MILLAR, J.L., BLACKETT, N.M. & HUDSPITH, B.N. (1978*a*). Enhanced post irradiation recovery of the haemopoietic system in animals pretreated with a variety of cytotoxic agents. *Cell Tissue Kinet.*, **11**, 543.

MILLAR, J.L., HUDSPITH, B.N., McELWAIN, T.J. & PHELPS, T.A. (1978*b*). Effect of high-dose melphalan on marrow and intestinal epithelium in mice pretreated with cyclophosphamide. *Br. J. Cancer*, **38**, 137.

MILLAR, J.L., PHELPS, T.A., CARTER, R.L. & McELWAIN, T.J. (1978*c*). Cyclophosphamide pretreatment reduces the toxic effect of high dose melphalan on intestinal epithelium in sheep. *Eur. J. Cancer*, **14**, 1283.

MURRAY, S.L., DuVALL, E.M. & SLATER, L.M. (1984). Calcium modifies the accumulation and retention of daunorubicin by Ehrlich ascites carcinoma. *Cancer Chemother. Pharmacol.*, **13**, 69.

MYERS, C.D., KATZ, F.E., JOSHI, G. & MILLAR, J.L. (1984). A cell line secreting stimulating factors for CFU-GEMM culture. *Blood*, **64**, 152.

PRITCHARD, J., McELWAIN, T.J. & GRAHAM-POLE, J. (1982). High-dose melphalan with autologous marrow for treatment of advanced neuroblastoma. *Br. J. Cancer*, **42**, 86.

RAMU, A., GLAUBIGER, D. & WEINTRAUB, H. (1984). Differences in lipid composition of doxorubicin-sensitive and resistant P388 cells. *Cancer Treat. Rep.*, **68**, 637.

REDWOOD, W.R. & COLVIN, M. (1980). Transport of melphalan by sensitive and resistant L1210 cells. *Cancer Res.*, **40**, 1144.

ROGAN, A.M., HAMILTON, T.C., YOUNG, R.C., KLECKER, R.W. & OZOLS, R.F. (1984). Reversal of adriamycin resistance by verapamil in human ovarian cancer. *Science*, **224**, 994.

SIMPSON, W.G., TSENG, M.T., ANDERSON, K.C. & HARTY, J.I. (1984). Verapamil enhancement of chemotherapeutic efficacy in human bladder cancer cells. *J. Urology*, **132**, 574.

SKOVSGAARD, T., DANO, K. & NISSEN, N.I. (1984). Chemosensitizers counteracting acquired resistance to anthracyclines and vinca alkaloids *in vivo*. A new treatment principle. *Cancer Treat. Rev.*, **11**, (Suppl A), 63.

SLATER, L.M., MURRAY, S.L., WETZEL, M.W., WISDOM, R.M. & DuVALL, E.M. (1982). Verapamil restoration of daunorubicin responsiveness in daunorubicin-resistant Ehrlich ascites carcinoma. *J. Clin. Invest.*, **70**, 1131.

TILL, J.E. & McCULLOCH, E.A. (1961). A direct measurement of the radiation sensitivity of normal mouse bone marrow cells. *Radiat. Res.*, **14**, 213.

TSURUO, T., IIDA, H., TSUKAGOSHI, S. & SAKURAI, Y. (1981). Overcoming of vincristine resistance in P388 leukaemia *in vivo* and *in vitro* through enhanced cytotoxicity of vincristine and vinblastine by verapamil. *Cancer Res.*, **41**, 1967.

TSURUO, T., IIDA, H., TSUKAGOSHI, S. & SAKURAI, Y. (1982). Increased accumulation of vincristine and adriamycin in drug-resistant P388 tumor cells following incubation with calcium antagonists and calmodulin inhibitors. *Cancer Res.*, **42**, 4730.

TSURUO, T., IIDA, H., NAGANUMA, K., TSUKAGOSHI, S. & SAKURAI, Y. (1983). Promotion by verapamil of vincristine responsiveness of tumor cell lines inherently resistant to the drug. *Cancer Res.*, **43**, 808.

VISTICA, D.T. (1980). Cytotoxicity as an indicator for transport mechanism: Evidence that murine bone marrow progenitor cells lack a high-affinity leucine carrier that transports melphalan in murine L1210 cells. *Blood*, **56**, 427.

VISTICA, D.T. (1983). Cellular pharmacokinetics of the phenylalanine mustards. *Pharmac. Ther.*, **22**, 379.

VISTICA, D.T., FULLER, R., DILLON, N. & PETRO, B.J. (1983). Comparative reactivity of cyclic amino acids with system L in murine L1210 leukaemia cells and murine bone marrow progenitor cells (CFU-C): A potential basis for selective drug design. In *Rational Basis for Chemotherapy*, Chabner, B.A. (ed) p. 475. Alan R. Liss, New York.

WITHERS, H.R. & ELKIND, M.M. (1970). Microcolony survival assay for cells of mouse intestinal mucosa exposed to radiation. *Int. J. Radiat. Biol.*, **17**, 261.

YALOWICH, J.C. & ROSS, W.E. (1984). Potentiation of etoposide-induced DNA damage by calcium antagonists in L1210 cells *in vitro*. *Cancer Res.*, **44**, 3360.

YALOWICH, J.C. & ROSS, W.E. (1985). Verapamil-induced augmentation of etoposide accumulation in L1210 cells *in vitro*. *Cancer Res.*, **45**, 1651.

YANOVICH, S. & PRESTON, L. (1984). Effects of verapamil on daunomycin cellular retention and cytotoxicity in P388 leukaemic cells. *Cancer Res.*, **44**, 1743.